# Antioxidant Therapy as a Potential Approach to Severe Influenza-Associated Complications

**DOI:** 10.3390/molecules23100000

**Published:** 2011-02-28

**Authors:** Noboru Uchide, Hiroo Toyoda

**Affiliations:** Department of Clinical Molecular Genetics, School of Pharmacy, Tokyo University of Pharmacy and Life Sciences, 1432-1 Horinouchi, Hachioji, Tokyo 192-0392, Japan

**Keywords:** antioxidant, influenza, antiviral drug

## Abstract

With the appearance of the novel influenza A (H1N1) virus 2009 strain we have experienced a new influenza pandemic and many patients have died from severe complications associated with this pandemic despite receiving intensive care. This suggests that a definitive medical treatment for severe influenza-associated complications has not yet been established. Many studies have shown that superoxide anion produced by macrophages infiltrated into the virus-infected organs is implicated in the development of severe influenza-associated complications. Selected antioxidants, such as pyrrolidine dithiocabamate, *N*-acetyl-l-cysteine, glutathione, nordihydroguaiaretic acid, thujaplicin, resveratrol, (+)-vitisin A, ambroxol, ascorbic acid, 5,7,4-trihydroxy-8-methoxyflavone, catechins, quercetin 3-rhamnoside, iso- quercetin and oligonol, inhibit the proliferation of influenza virus and scavenge superoxide anion. The combination of antioxidants with antiviral drugs synergistically reduces the lethal effects of influenza virus infections. These results suggest that an agent with antiviral and antioxidant activities could be a drug of choice for the treatment of patients with severe influenza-associated complications. This review article updates knowledge of antioxidant therapy as a potential approach to severe influenza-associated complications.

## 1. Introduction

With the appearance of the novel influenza A (H1N1) virus 2009 strain we have recently experienced a new influenza pandemic [1,2]. The clinical spectrum of pandemic influenza A (H1N1) virus infection was broad, ranging from mild upper respiratory tract illness with or without fever and occasional gastrointestinal symptoms such as vomiting or diarrhea and exacerbation of underlying conditions, to severe complications such as pneumonia resulting in respiratory failure, acute respiratory distress syndrome, multi-organ failure and even death [3,4]. Many patients died from severe complications associated with the pandemic influenza A (H1N1) virus infection despite receiving intensive care [3,4], and as of the 25^th^ of July 2010, 18,398 laboratory-confirmed fatal cases of pandemic influenza A (H1N1) have been reported to the World Health Organization [5]. The influenza A (H1N1) virus human infections event has now moved into a post-pandemic period, with a pattern that has been transitioning towards that of seasonal influenza [6]. Beside the influenza A (H1N1) pandemic the global burden of seasonal influenza epidemics is believed to be some 3-5 million cases of severe illness and 300,000–500,000 deaths every year [7]. Additionally, we still face the threat of infection with the highly pathogenic avian influenza A (H5N1) virus.

**Figure 1:**
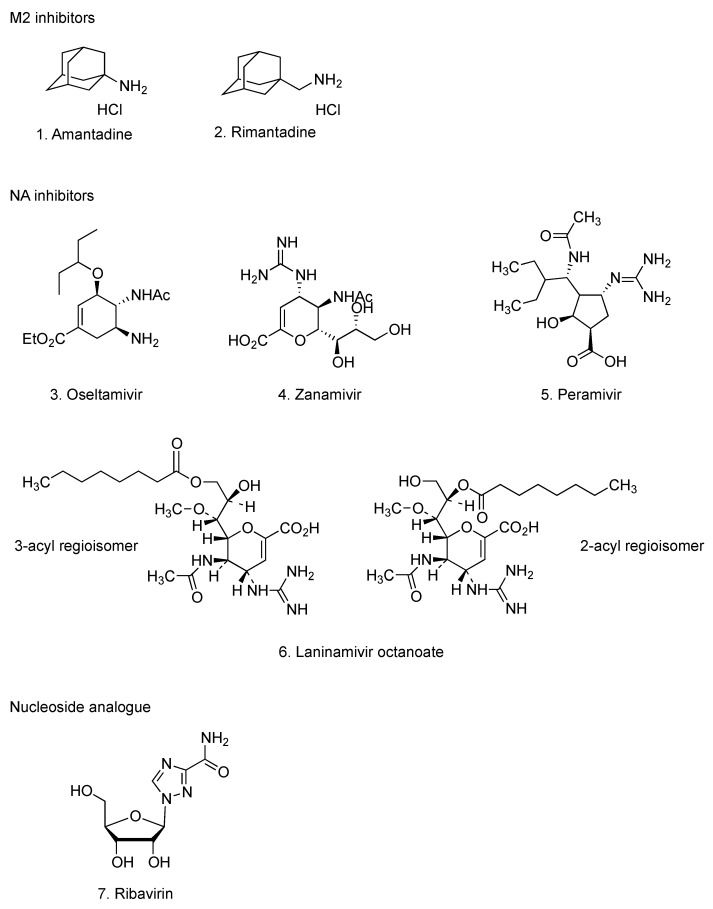
Current available anti-influenza drugs.

Three classes of anti-influenza drugs have been used for chemoprophylaxis and treatment of influenza virus infections [8] (Figure 1): amantadine (**1**) and rimantadine (**2**) which inhibit viral membrane protein (M2) of the proton channel that is necessary for uncoating; oseltamivir (**3**), zanamivir (**4**), peramivir (**5**) and laninamivir octanoate (**6**) which inhibit viral neuraminidase (NA) that is necessary for virion release and ribavirin (**7**) that inhibits enzyme activity essential for viral replication. Initial diagnostic testing found that the pandemic influenza A (H1N1) virus was susceptible to NA inhibitors, but resistant to M2 inhibitors [9], therefore, oseltamivir has been used widely for treatment and chemoprophylaxis of pandemic influenza A (H1N1) [2]. Sporadic cases of oseltamivir-resistant pandemic influenza A (H1N1) virus have been reported worldwide [10]. This oseltamivir resistance was caused by the NA mutation H275Y [10]. Person-to-person transmission of oseltamivir-resistant viruses in healthy adults has been confirmed [11]. In cases of development of oseltamivir-resistance, treatment options are limited because zanamivir is not licensed for treatment of children under 7 years old and is contraindicated in persons with underlying airway disease. Recently, it has been reported that a single inhalation of laninamivir octanoate was an effective and well-tolerated drug for the treatment of children with oseltamivir-resistant influenza A (H1N1) virus infection [12]. Additionally, intravenous drip infusion of peramivir has offered a new treatment option for children and infants suffering from influenza virus infections and patients where oral administration was difficult or not possible [13]. It was also effective for severe influenza-associated complications, such as acute respiratory failure [14]. Nonetheless, NA inhibitor-resistant viruses with H275Y mutation emerged early and replicated in patients, who have received hematopoietic cell transplant, under treatment with immunosuppressive drugs after intravenous drip infusion of peramivir [15]. A young adult with pandemic influenza A (H1N1) virus infection was treated with intravenous peramivir, but died from severe viral pneumonia [16]. These results suggest the need for development of new anti-influenza drugs utilizing alternative antiviral mechanisms and consideration of using anti-influenza drug combinations. Some such approaches have been explored, whereby a triple combination of amantadine, ribavirin and oseltamivir was highly active and synergistic against drug resistant influenza virus strains *in vitro* [17]. 

**Figure 2:**
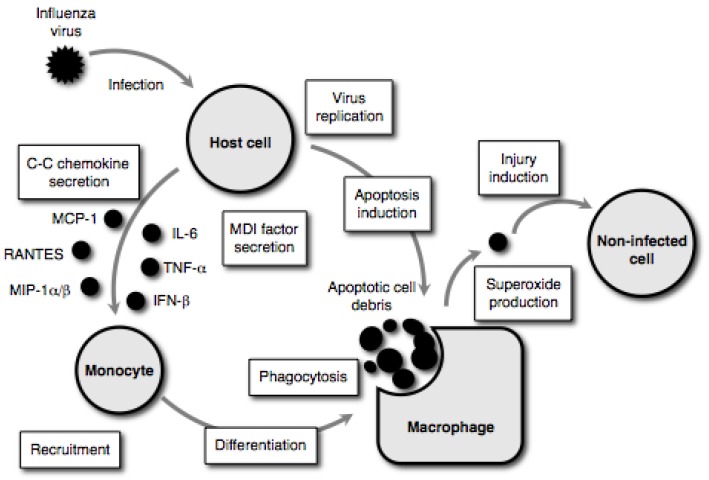
Tissue injury model during influenza virus infection.

In cases of severe influenza-associated complications, the pathological manifestations are the result of complex biological phenomena, such as apoptosis induction, macrophage activation, oxidative tissue damage and higher contents of pro-inflammatory cytokines [18]. The pathogenesis of severe influenza-associated complications involves not only apoptotic cell death mediated through virus replication in the infected cells, but also the injury of non-infected cells by superoxide anion derived from activated phagocytes (*i.e.*, macrophages and neutrophils) infiltrated into the virus-infected organs [19]. As illustrated in Figure 2 [19], host cells secrete cysteine-cysteine (C-C) chemokines that primarily target monocytes [e.g., monocyte chemoattractant protein (MCP)-1, regulated on activation, normal T cell expressed and secreted (RANTES) and macrophage inflammatory protein (MIP)-1α/β) and monocyte differentiation-inducing (MDI) factor (*i.e.*, interleukin (IL)-6, tumor necrosis factor (TNF)-α and interferon (IFN)-β] in response to influenza virus replication prior to undergoing apoptotic cell degradation. The C-C chemokines act on immature monocytes circulating in the bloodstream, recruiting them to the site of infection. The MDI factor acts on the recruited monocytes, resulting in differentiation into well-matured macrophages capable of phagocytosing and producing superoxide anion. The activated macrophages move to the virus- infected host cell and phagocytose apoptotic cell debris resulting from the viral infection. An abrupt increase in superoxide anion production occurs during phagocytosis. This superoxide anion induces injuries in non-infected cells. These superoxide anion-mediated pathways represent a part of the mechanisms of extensive tissue injury observed during severe influenza-associated complications [20], therefore, it has been suggested that an agent with antiviral and antioxidant activities could be a drug of choice for the treatment of patients with such severe complications [21,22]. This review article updates knowledge of antioxidant therapy as a potential approach to these severe influenza-associated complications.

## 2. Effect of Selected Antioxidants on the Pathogenesis of Influenza Virus Infections

### 2.1. Superoxide dismutases

Intravenous injection of pyran polymer conjugated with copper/zinc (Cu/Zn)-superoxide dismutase (SOD) protected mice against a potentially lethal influenza virus infection [23]. Intravenous injection of manganese (Mn)-SOD to mice with influenza virus infection at a lethal dose mildly increased mean days of survival, lessened arterial oxygen saturation decline, and lowered lung consolidation [24]. A combination of Mn-SOD and ribavirin, each of which was administered as a small-particle aerosol, resulted in a generally mild improvement of the disease induced by the influenza A virus, compared with use of either substance alone [24]. A combined application of Cu/Zn-SOD and rimantadine hydrochloride in doses which by themselves did not protect significantly mice against the infection, resulted in a synergistic decrease in lung virus titers, lung weights and consolidation and mortality rates [25]. Treatment with allopurinol, an inhibitor of xanthine oxidase capable of generating superoxide anion, improved the survival rate of influenza virus-infected mice [26]. It should be noted that the treatment with SODs decreased the lethal or toxic effect of influenza virus infection in mouse models, but did not inhibit the virus proliferation [23,24,25,26]. Transgenic mice carrying overexpressed extracellular SOD exhibited less severe lung injury after influenza virus infection [27]. Mice lacking functional phagocyte NADPH oxidase capable of generating superoxide anion exhibited an increase of macrophages and the reduction of apoptosis in macrophages and virus titer in the bronchoalveolar space after influenza virus infection, as compared to those of wild-type animals [28]. Therefore, these studies suggest that superoxide anion produced by phagocytes, especially macrophages, plays a critical role in the pathogenesis of influenza virus infections. Since SODs do not inhibit the proliferation of influenza virus, an agent possessing both antiviral and antioxidant activities would be desirable for antioxidant therapy as a new approach to severe influenza-associated complications.

### 2.2. Thiol antioxidants

#### 2.2.1. Pyrrolidine dithiocarbamate

**Figure 3:**
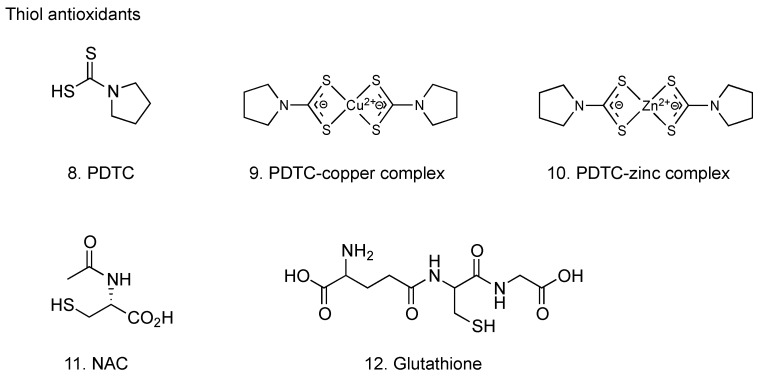
Thiol antioxidants with anti-influenza virus activity.

Pyrrolidine dithiocarbamate (PDTC, **8**, Figure 3) has been shown to scavenge hydroxyl and superoxide anion radicals; its effect is comparable to that of other free radical scavengers, such as ascorbate and glutathione [29]. Both PDTC and Trolox^®^ (6-hydroxy-2,5,7,8-tetra- methylchroman-2-carboxylic acid; a water-soluble vitamin E analogue) suppressed the accumulation of reactive oxygen species (ROS) in chorion cells caused by influenza virus infection [30,31]. PDTC inhibited both apoptosis induction and virus proliferation in the infected chorion cells, whereas no such inhibitory effect was observed with Trolox^®^ [30,31]. PDTC also inhibited the cytopathic effect of influenza virus infection on the other types of cells, such as human pulmonary epithelial A549 cells and murine macrophage J774.1 cells [32,33,34]. Studies using J774.1 cells also demonstrated that various other antioxidants, such as Trolox^®^, deferoximine nesyiate, dithiothreitol, *N*-methyl-D-arginine, catalase and SOD, did not inhibit the cytopathic effects of influenza virus infection [33,34]. These results suggest that the inhibition of influenza virus-induced apoptosis by PDTC is attributable to its antiviral activity rather than its antioxidant properties. That is, ROS may not be responsible for apoptosis induction by influenza virus infection [30,31]. However, as described in the previous section, it has been suggested that organ injury was occurred by ROS derived from phagocytes during influenza virus infection in mouse models. Therefore, these evidence reveals that the pathogenesis of influenza virus infection involves not only the virus replication-mediated apoptotic cell death in the infected cells irrespective of ROS, but also the injury of non-infected cells by ROS derived from macrophages and neutrophils infiltrated into the virus-infected organs. The findings support the notion that an agent with antiviral and antioxidant activities can be a drug of choice for the treatment of patients with severe influenza-associated complications and that PDTC could be one of these drug candidates. Since PDTC is well tolerated *in vivo* at doses of up to 100 mg/kg (intraperitoneal injection) and exhibits therapeutic effects on animal inflammation and tissue injury models [35,36,37,38], further studies on the therapeutic effect of PDTC on animal influenza models are warranted.

The mode of inhibitory effect of PDTC on influenza virus proliferation has been investigated. PDTC inhibited the synthesis of negative-strand virion RNA (vRNA) and positive-strand complementary and/or messenger RNA (c/mRNA) for influenza virus HA gene in the infected cells [31], therefore it is likely that the inhibition of influenza virus gene replication and transcription contributes to the inhibition of virus proliferation. Dithiocarbamates can chelate various divalent metal ions, leading to the formation of lipophilic dithiocarbamate-metal complexes, and rapid transport via a lipophilic complex by PDTC has been proposed to explain the intracellular recruitment of copper and zinc ions from the extracellular medium [39]. It has been demonstrated that copper and zinc ions inhibit influenza virus RNA-dependent RNA polymerase activity, and that the inhibitory effect of bathocuproine-copper and batho- cuproine-zinc complexes is greater than that of bathocuproine itself [40]. Moreover, it has been known that PDTC-copper and PDTC-zinc complexes (**9** and **10**, respectively, Figure 3) inhibited the replication of Coxsackie virus [41] and rhinovirus [42]. Conceivably, PDTC may inhibit influenza virus gene replication and transcription through the inhibition of viral RNA-dependent RNA polymerase activity by increasing the amount of intracellular copper and zinc ions or intracellular PDTC-copper and PDTC-zinc complexes, but further study is needed to elucidate the precise mechanism of inhibitory effect of PDTC on influenza virus gene replication and transcription.

Furthermore, it has been revealed that PDTC inhibited the induction of gene expression for MDI factors and C-C chemokines, such as IL-6, TNF-α, IFN-β and RANTES, by influenza virus infection [43,44, our unpublished data]. Consequently, PDTC may attenuate the activation of macrophages during influenza virus infection via a suppressive effect on production of cytokines, thus reducing the pathogenesis of influenza virus infections.

#### 2.2.2. *N*-Acetyl-l-cysteine

*N*-Acetyl-l-cysteine (NAC, **11**, Figure 3), the acetylated derivative of the amino acid l-cysteine, is an excellent source of thiol groups, and is converted in the body into metabolites capable of stimulating glutathione synthesis, promoting detoxification, and acting directly as free radical scavengers [45]. NAC inhibited the induction of apoptosis [46,47,48] and gene expression for pro-inflammatory cytokines and chemokines such as IL-6, IL-8, RANTES and interferon-inducing protein (IP)-10, by influenza virus infection [48]. NAC inhibited the proliferation of influenza virus at an early, but not later, stage of infection [47,48]. Administration of NAC significantly decreased the mortality in mice infected with influenza virus [49], and a combination of NAC and ribavirin synergistically reduced the lethal effects [50]. Furthermore, a combination of NAC and oseltamivir also synergistically reduced the lethal effect of influenza virus infection in mice [51]. These results support the notion that combinations of antioxidant therapy with current drugs can improve the treatment of influenza virus infections.

Administration of NAC appears to reduce symptomatic conditions associated with influenza virus infection. A total of 262 subjects of both sexes were given either placebo or NAC (600 mg) orally twice daily for six months. Although incidents of seroconversion towards A (H1N1) Singapore 6/86 influenza virus was similar in the two groups, NAC treatment decreased both the incidence and severity of influenza-like episodes, and the length of time confined to bed. The authors concluded that NAC did not prevent influenza A (H1N1) virus infection, but did significantly reduce the incidence of clinically apparent disease [52]. In another paper, it has been reported that a patient with viral pneumonia caused by the novel influenza A (H1N1) virus 2009 infection and septic shock improved rapidly after continuous intravenous infusion of high-dose NAC at 100 mg/kg combined with oseltamivir [53]. 

#### 2.2.3. Glutathione

Reduced glutathione (**12**, Figure 3) displays anti-influenza activity *in vitro* and *in vivo* [54]. The addition of reduced glutathione into culture medium exogenously blocked the induction of apoptosis through the inhibition of viral macromolecule synthesis in Madin-Darby canine kidney (MDCK) cells after influenza virus infection. The antiviral effect of reduced glutathione on influenza virus proliferation was also observed in normal human small airway epithelial cells. In BALB/c mice, inclusion of reduced glutathione in the drinking water decreased viral titer in both lung and trachea homogenates at 4 days after intranasal inoculation with a mouse-adopted influenza strain A/X-31. Moreover, both the levels of Bcl-2 expression and the content of intracellular reduced glutathione contribute to the ability of host cells for down-regulating influenza virus replication, although their effects are exerted at different stages of the viral life-cycle [55].

### 2.3. Hydroxyl antioxidants

#### 2.3.1. Nordihydroguaiaretic acid

**Figure 4:**
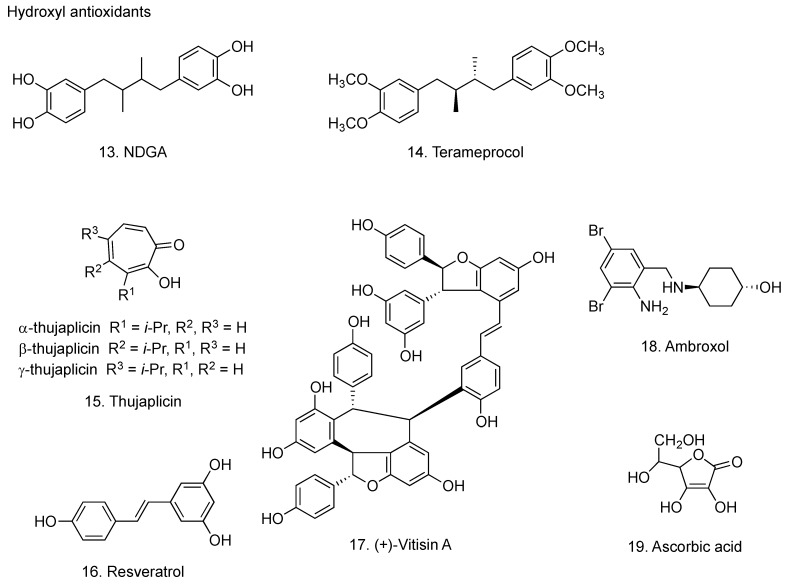
Hydroxyl antioxidants with anti-influenza virus activity.

Nordihydroguaiaretic acid [1,4-bis(3,4-dihydroxyphenyl)-2,3-dimethylbutane, NDGA, **13**, Figure 4] occurs in the resinous exudates of the creosote bush *Larrea divaricata*. NDGA scavenges oxygen radicals, such as peroxynitrite, singlet oxygen, hydroxyl and superoxide anion radicals [56]. The ability of NDGA to scavenge the above-mentioned oxygen radicals is much greater than that of reference tested compounds, such as uric acid, penicillamine, reduced glutathione and mannitol [56]. These strong antioxidant properties may be due to the presence of four reducing equivalents from the two catechol groups in NDGA; hydrogen atoms of the four phenolic hydroxyl groups react with oxygen radicals [56]. NDGA has been shown to have promising applications in the treatment of multiple diseases, including cardiovascular diseases, neurological disorders, cancers and virus infections [57]. The treatment with NDGA inhibited apoptotic DNA fragmentation and virus proliferation in chorion cells infected with influenza virus [58]. Erimos Pharmaceuticals, in collaboration with researchers at North Carolina State University (NCSU), has shown potential benefits of terameprocol (tetra-*O*-methyl NDGA, **14** Figure 4) [59] in treating pneumonia and other symptoms related to influenza virus infection. Eads and co-workers in NCSA have demonstrated the inhibitory effect of terameprocol on prostaglandin E_2_ production in macrophages infected with influenza virus [60].

Recently, it has been reported that several methylated derivatives of NDGA possessed an inhibitory activity on the expression of reporter genes driven by some viral promoters of herpes simplex virus, human papillomavirus and human immunodeficiency virus, which was resulting from the inhibitory effect on the binding of cellular transcription factor Sp-1 to viral gene promoters [61]. 

NDGA derivatives did not affect the expression of reporter genes driven by the adenovirus major late promoter and the cytomegalovirus promoter [61]. It is predicted that the antiviral activity of NDGA derivatives is selective, depending on the virus types. Influenza virus has viral RNA-dependent RNA polymerases, which contribute to the replication and transcription processes of the viral genes, probably irrespective of cellular transcription factor Sp-1. An additional mechanism of NDGA for the inhibition of influenza virus proliferation has been proposed. NDGA is shown to inhibit the intracellular transport of vesicular stomatitis virus glycoproteins [62]. Conceivably, NDGA may inhibit influenza virus proliferation via inhibition of intracellular transport of viral glycoproteins.

#### 2.3.2. Thujaplicin

The thujaplicins, including α-thujaplicin (2-hydroxy-3-isopropyl-2,4,6-cycloheptatrien-1- one), β-thujaplicin (2-hydroxy-4-isopropyl-2,4,6-cycloheptatrien-1-one) and γ-thujaplicin (2-hydroxy-5-isopropyl-2,4,6-cycloheptatrien-1-one) (**15**, Figure 4), are tropolone-related compounds found in the heartwood of several cupressaceous plants, such as Western Red Cedar (*Thuja plicata*), Eastern White Cedar (*Thuja occidentalis*) and Hinoki Cypress (*Chamaecryprais obtusa*) [63]. Copper complexes of α-, β- and γ-thujaplicin blocked the induction of apoptosis in MDCK cells by influenza virus infection through their antiviral effects, while their ferrous, ferric, magnesium and manganese complexes showed no inhibition of influenza virus-induced apoptosis [64]. Thujaplicin scavenges hydroxyl radical, *tert*-butyl peroxyl radical, hydrogen peroxide, superoxide anion radical and singlet oxygen [65].

#### 2.3.3. Resveratrol

The plant polyphenol resveratorol (3,5,4’-trihydroxy-*trans*-stilbene) (**16**, Figure 4) inhibited the progressive effects of superoxide anion and hydrogen peroxide radicals on arachidonic acid production and cyclooxygenase-2 induction in macrophages [66]. Resveratrol inhibited the replication of influenza virus in MDCK cells, as a result of the blockade of the nuclear- cytoplasmic translocation of viral ribonucleoproteins and the reduced expression of late viral protein, such as HA and matrix protein [67]. Resveratol also improved survival and decreased pulmonary viral titers in influenza virus-infected mice [67]. Since resveratrol inhibited the induction of RANTES production by influenza virus infection in A549 lung epithelial cells [68], it is presumed that resveratorol attenuate the activation of macrophages during influenza virus infection.

(+)-Vitisin A (**17**, Figure 4), a tetramer of resveratrol, isolated from *Vitis thunbergii* was reported to have various bioactivities, including antioxidant, protection against platelet aggregation, to inhibit the biosynthesis of pro-inflammatory cytokine leukotriene B4, and to suppress TNF-α-induced monocyte chemoattractant protein production in primary human endothelial cells [69,70,71]. Furthermore, (+)-vitisin A has been shown to inhibit the production of RANTES in airway epithelial cells after influenza A virus infection, the effect of which was much higher than that of resveratrol [72]. Accordingly, it has been suggested that (+)-vitisin A may serve as a potential anti-inflammatory agent that interrupts the pathogenesis after viral infection.

#### 2.3.4. Ambroxol

Ambroxol (2-amino-3,5-dibromo-*N*-[trans-4-hydroxycyclohexyl]benzylamine, **18**, Figure 4), known as a mucolytic agent, has been used for the treatment of chronic bronchitis and neonatal respiration distress syndrome [73]. Ambroxol suppressed the proliferation of influenza virus in the mouse airway and improved the survival rate of mice [74]. Antioxidant activity of ambroxol is related to the direct scavenging effect for ROS, such as superoxide anion and hydroxyl radicals [75,76]. Treatment with ambroxol also significantly decreased the incidence of acute upper respiratory diseases during winter season in humans [77].

#### 2.3.5. Ascorbic acid

Ascorbic acid (**19**, Figure 4) scavenges superoxide anion [78]. Ascorbic acid inhibited the proliferation of influenza virus in cell cultures [79]. Dehydroascorbic acid, an oxidized form of ascorbic acid without reducing ability, showed much stronger antiviral activity than that of ascorbic acid, indicating that the antiviral activity of ascorbic acid is due to factors other than antioxidant mechanism [80]. In a controlled trial of 226 patients with influenza A, 114 patients received vitamin C 300 mg/day, and 112 patients served as controls; outcomes measured were development of pneumonia and duration of hospital stay. Pneumonia was reported in two subjects in the treatment group and 10 in the control group, and hospital stays for influenza or related complications averaged nine days in the vitamin C group and 12 days in the control group [81]. Therefore it has been considered that combined inhalation and oral supplementation of ascorbic acid may prevent influenza virus infection [82].

### 2.4. Flavonoids

Flavonoids are a ubiquitous group of polyphenolic substances which are present in most plants, concentrating in seeds, fruit skin or peel, bark, and flowers. The structural components common to these molecules include two benzene rings on either side of a 3-carbon ring. Multiple combinations of hydroxyl groups, sugars, oxygens, and methyl groups attached to these structures create the various classes of flavonoids: flavanols, flavanones, flavones, flavan-3-ols (catechins), anthocyanins, and isoflavones. Flavonoids have been shown in a number of studies to be potent antioxidants, capable of scavenging hydroxyl radicals, superoxide anions, and lipid peroxyl radicals [83].

#### 2.4.1. 5,7,4’-Trihydroxy-8-methoxyflavone

**Figure 5:**
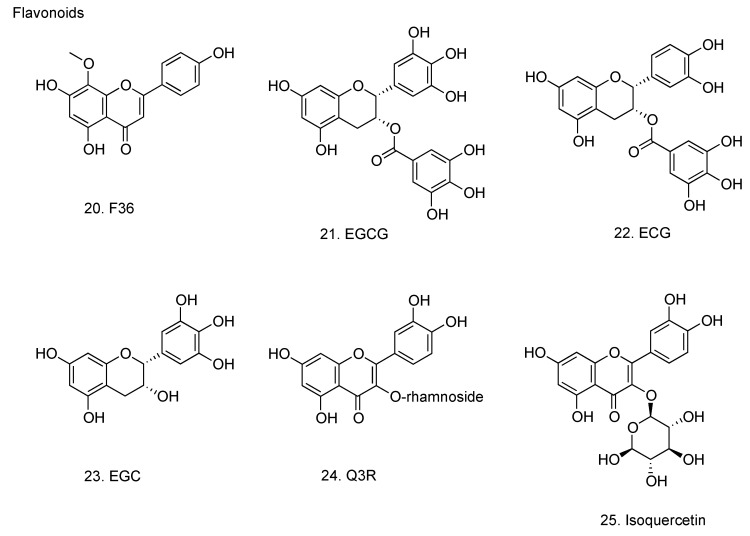
Flavonoids with anti-influenza virus activity.

5,7,4’-Trihydroxy-8-methoxyflavone (F36, **20**, Figure 5) isolated from the roots of *Scutellaria baicalensis*, was shown to have a specific inhibitory activity against influenza virus NA because it did not affect the mouse liver NA activity [84,85]. F36 inhibited the proliferation of influenza virus in MDCK cells, in the allantoic sac of embryonal chicken egg and *in vivo* using BALB/c mice [85,86,87]. Immunoelectron microscopic analysis revealed that F36 inhibited the budding of progeny influenza virus particles from MDCK cell surface and microvilli [88].

#### 2.4.2. Catechins

Catechins from green tea: (-)-epigallocatechin gallate (EGCG), (-)-epicatechin gallate (ECG) and (-)-epigallocatechin (EGC) (**21**, **22** and **23**, respectively, Figure 5) have been evaluated for their ability to inhibit influenza virus replication in cell culture [89]. Among the test compounds, EGCG and ECG were found to be potent inhibitors of influenza virus replication in MDCK cell culture, and this effect was observed in all influenza virus subtypes tested, including A (H1N1), A (H3N2) and B virus. The 50% effective inhibition concentrations of EGCG, ECG, and EGC for influenza A virus were 22–28, 22–40 and 309–318 µM, respectively. EGCG and ECG exhibited inhibitory activity of hemagglutination, suppressed viral RNA synthesis in MDCK cells, and inhibited the NA activity, however, the effects of EGC were much lesser. The results show that the 3-galloyl group of catechin skeleton plays an important role on the observed antiviral activity, whereas the 5’-OH at the trihydroxybenzyl moiety at the 2-position plays a minor role. Catechins have been shown to possess the ability to scavenge for superoxide anion and hydroxyl radicals [90]. Gargling with tea catechin extracts prevented influenza virus infection in elderly nursing home residents [91]. Long chain monoester derivatives of EGCG enhanced the anti-influenza virus activity 24-fold relative to native EGCG [92].

The influenza A RNA polymerase possesses endonuclease activity to digest the host mRNA. This endonuclease domain can be a target of anti-influenza A virus drug. Kizuhara and co-workers have reported that green tea catechins inhibited this viral endonuclease activity and that their galloyl group was important for their function [93]. Docking simulations revealed that catechins with galloyl group fitted well into the active pocket of the endonuclease domain to enable stable binding. Their results provide useful data that make it possible to refine and optimize catechin-based drug design more readily for stability.

#### 2.4.3. Quercetin 3-rhamnoside

Quercetin 3-rhamnoside (Q3R, 24, Figure 5) from *Houttuynia cordata* possessed strong antiviral activity against influenza A/WS/33 virus as well as oseltamivir [94]. Pre-exposure of the virus to Q3R did not alter the infectivity. When Q3R was added just after the virus infection or until four hours after the virus infection, the antiviral activity of Q3R was exhibited. Viral mRNA synthesis was inhibited by the treatment with Q3R. The mode of action of Q3R involved the inhibition of virus replication in the initial stage of virus infection by indirect interaction with virus particles.

#### 2.4.4. Isoquercetin

Isoquercetin (**25**, Figure 5) inhibited the replication of both influenza A and B viruses in cell cultures, the antiviral activity of which was much stronger than that of EGCG, resveratrol and quercetin [95]. Combination of isoquercetin with amantadine synergistically reduced the viral replication *in vitro*. The serial passages of virus in the presence of isoquercetin did not lead to the emergence of resistant virus, and the addition of isoquercetin to amantadine or oseltamivir treatment suppressed the emergence of amantadine- or oseltamivir-resistant virus. In a mouse model of influenza virus infection, isoquercetin administered intraperitoneally to mice inoculated with human influenza A virus significantly decreased the virus titers and pathological changes in the lung. Therefore, it has been suggested that isoquercetin may have the potential to be developed as a therapeutic agent for the treatment of influenza virus infection and for the suppression of resistance in combination therapy with existing drugs.

#### 2.4.5. Oligonol

Oligonol was obtained by oligomerizing the polyphenol polymers found in lychee fruit pericarp [96]. As indicated in Table 1, oligonol contains 16.1% polyphenol monomers [(+)-catechin, (-)-epicatechin, ECG and EGCG] and 13.9% polyphenol dimer (procyanidin A1 *etc.*), while lychee fruit polyphenol contains 6.4% polyphenol monomer and 9.9% polyphenol dimer [97]. 

Oligonol was approved as a New Dietary Ingredient by the United States Food and Drug Administration in 2007, and is commercially available at present (Amino Up Chemical Co., Ltd., Sapporo, Japan). Oligonol inhibits influenza virus proliferation by blocking attachment of the virus to MDCK cells and by suppression of nuclear export of influenza virus ribonucleoprotein (RNP) [97]. Influenza virus infection induced the production of ROS and the phosphorylation of extracellular-signal-regulated kinase (ERK) in MDCK cells. Inhibition of ERK activation by a dominant negative mutant of ERK or NAC led to the suppression of viral RNP nuclear export. Phorbol 12-myristate 13-acetate (PMA) induced ROS production, ERK phosphorylation and enhanced influenza proliferation in MDCK cells. Oligonol and NAC inhibit PMA-induced ERK phosphorylation and ROS production. These results suggest that the inhibitory effect of oligonol on influenza virus RNP nuclear export is implicated in the blocking of ROS-dependent induction of ERK phosphorylation.

## 3. Conclusions

Scavenging of superoxide is an important tool in the development of new strategies for the prevention of organ failure during severe influenza-associated complications. Since the most important aspect in viral disease treatment is to inhibit virus replication, an agent with antiviral and antioxidant activities should be a drug of choice for the treatment of patients with severe influenza-associated complications. Selected compounds, such as PDTC, NAC, glutathione, NDGA, thujaplicin, resveratrol, (+)-vitisin A, ambroxol, ascorbic acid, F36, EGCG, ECG, Q3R, isoquercetin and oligonol, possess both antiviral and antioxidant activities. Consequently, they are potential drugs of interest for severe influenza-associated complications. In theory, combination of these antioxidants with current anti-influenza drugs could improve conventional chemotherapy for severe influenza-associated complications.

## Figures and Tables

**Table 1:** Composition of oligonol and lychee fruit polyphenol.

	Name	Oligonol	Lychee fruit polyphenol
Monomer	(+)-Catechin	0.1%>	0.1%>
	(-)-Epicatechin	7.5%	6.4%
	(-)-ECG	2.1%	n.d.
Dimer	Procyanidin A1	4.2%	4.0%
	Procyanidin A2	5.1%	3.3%
	Procyanidin B1	1.4%	0.8%
	Procyanidin B2	2.9%	1.7%
Trimer	(-)-Epicatechin-(4β→8,2β→*o*-7)-epicatechin-(4β→8)-epicatechin	4.0%	3.6%

Abbreviation: n.d., not detected.
